# “Caregiving Youth” and the Patchwork History of Recognition in the United States

**DOI:** 10.3390/ijerph20206920

**Published:** 2023-10-13

**Authors:** Elizabeth Olson, Leiha Edmonds

**Affiliations:** Department of Geography, University of North Carolina at Chapel Hill, Chapel Hill, NC 27599, USA; ledmonds@unc.edu

**Keywords:** caregiving youth, history, United States, feminist geography, care, critical health geographies

## Abstract

This article examines the U.S. legislative and policy landscape and its historical and contemporary recognition of young people as caregivers and their importance to public health, both as care providers and as a category of special concern for overall wellbeing. Drawing on feminist geographies of health to situate a historical analysis, we aim to answer two key questions: First, what is the history of recognition of caregiving youth in key moments of federal action to address family caregiving needs? Second, how might we use this history to better understand and analyze the patchwork geography of caregiving youth recognition in the U.S. and other countries that similarly lack formal national policy recognition to improve and enhance public health? We use the term patchwork to describe how federal recognition of caregiving youth in broader debates about public health is uneven across both time and space, and contingent upon civil society, non-profit organizations, and researchers working in and with geographically bound communities. Our results illustrate how a focus on the relationships of recognition, both in the past and the present and at local and national scales, reveals a different perspective on caregiving youth in the U.S. with a much more complex history than previously identified. The article describes how relationships established in the absence of federal policy or legislation are sometimes directed towards building more formal recognition, and other times with the goal of changing practices in a specific location.

## 1. Introduction

Children and adolescents remain largely unrecognized participants in the informal, unwaged family caregiving that millions of U.S. residents undertake daily to sustain their family members [1]. While a growing number of European, Asian, and African countries recognize that the concerns and experiences of youth caregivers are distinct from both older caregivers and their non-caregiving peers, the U.S. has been comparatively slow in moving from identification to further research or action [2,3]. The comparatively slow pace of public and legislative recognition of young carers in the U.S. contrasts a growing body of research on young carers in pediatrics [4], education and child development [5], social work [6], and the social sciences [7], which suggests the need for both more research and more interventions in support of caregiving families.

This article examines the U.S. legislative and policy landscape and its historical and contemporary recognition of young people as caregivers and their importance to public health, both as care providers and as a category of special concern for overall wellbeing. Though there is currently a diversity of federal and state legislation to support family caregivers as a broader concern of public health [8], the urgent attention paid to adult caregivers obscures the frustrated pace of caregiving youth recognition.

## 2. Methods and Research Objectives

In this paper, we expand on a body of research by feminist geographers who analyze care labor policy including England and Alcorn’s [9] analysis of the 1938 Fair Labor Standards Act (FLSA) and Lopez’s [10] study of both the 2017 Tax Cuts and Jobs Act and the 2010 Affordable Care Act. The article is not a systematic review, but instead relies upon qualitative and humanistic methods that include reviewing archival materials and published articles and monographs about the history and development of caregiver-focused policies in the U.S. The collected materials were analyzed to identify how people under the age of 18 are included or excluded in legislation and policy in key historical junctures. Our goal is to illustrate the importance of expanding how we theorize and account for recognition, to emphasize the importance of national histories and characteristics in both explaining and identifying support, and to detail the specific networks and collaborations that are changing how state and federal processes recognize caregiving youth in the U.S.

We aim to answer two key questions through this paper: First, what is the history of recognition of caregiving youth in key moments of federal action to address family caregiving needs? Second, how might we use this history to better understand and analyze the patchwork geography of caregiving youth recognition in the U.S. and other countries that similarly lack formal national policy recognition to improve and enhance public health? Reflecting on the role of young people who provide care to family members, we review how caregiving designation has been reinterpreted and redefined in the past 100 years while interrogating the limitations of the inclusion/exclusion binary framing legislation uses to categorize and understand care, caregivers, and recipients of care.

We begin our analysis by situating caregiving youth within a broader public health debate about the ‘care crisis’ in the U.S. Influenced by feminist geographers who draw attention to the ways in which power flows through and structures the interactions between environment and public health, we suggest that assessments of recognition can become too narrowly focused if limited to the formalization of rights while ignoring the processes through which ‘hidden’ populations become recognized in policy and practice. With this curiosity, and with the challenge posed by Scally and Womack [11] to enhance historical understandings of the emergence of public health movements, we reflect on three moments in which caregiving was a central preoccupation of public health policy and action in the U.S., beginning in the early industrial era, through the development of the Family Medical Leave Act, and into the first quarter of the 21st century, when caregiving youth have been formally recognized in federal legislation. We highlight how contemporary advocacy for caregiving youth has effectively challenged age-biased definitions of who is a caregiver in national policy. Our analysis suggests that the recognition of young people as caregivers in the U.S. has fluctuated over time and across space. Additionally, our analysis points to a range of factors that eventually lead to federal recognition, including state and local level programming, non-profit and research–practitioner partnerships, and community-driven innovations. We conclude by recommending that assessments of young carer/caregiving youth recognition should be more attendant to recognition across both time and space, not only because it offers a more robust understanding of both how and to what effect recognition of caregiving by young people can take root in national imaginaries of public health, even in the absence of national commitments to the rights of children.

## 3. Caregiving Youth and Recognition

For over a decade, caregiving advocates in the United States have raised concerns about the widening gap between the number of people who require caregiving and the availability of both professional and family caregivers [12]. Whereas past crises in care have been associated with the movement of women into the workforce [13], this current crisis is heightened by the transition of baby boomers into old age [14,15]. Research in gerontology [16], geography [17,18], and psychology and family studies [19] is particularly attentive to intergenerational families and grandparents raising grandchildren, but often focus on the roles of older adults as providers of care rather than consider interdependent care relationships where young people also give and receive care in their family. Cultural, racial, and ethnic diversity in the U.S. also reveals differences in the ways in which families make caregiving decisions, including their values about family care, the allocation of caregiving responsibilities, and racialized patterns of household poverty and health inequality [20]. Despite growing recognition of the implications of this aging crisis on the wellbeing of those who provide and need care in the U.S., youth caregivers remain largely unrecognized in this literature and excluded from research and legislation [21].

The National Academies of Sciences, Engineering, and Medicine [15] expert consensus report on family caregiving for aging America perfectly illustrates this exclusion; in what was heralded as a landmark report, children appear only as additional care burdens for ‘sandwich generations’ caring for aging parents while still raising younger children. Not surprisingly, research and policy directed toward youth caregivers in the U.S. have been deemed as stuck at an ‘emerging’ level [2,3], with limited national progress made in the past decade since the first national prevalence survey estimated there to be approximately 1.6 million youth caregivers in the U.S. [22].

The exclusion of youth caregiving from adult-focused caregiving research in the U.S. is especially notable when contrasted with scholarship abroad. Driven by a concern about the unmet needs of potentially vulnerable youth, particularly by experts in social work and legal studies, international research on youth caregiving has focused broadly on (a) identifying and characterizing the kinds of caregiving provided by children and adolescents [2], and (b) understanding the social impacts of caregiving upon youth [23]. In Europe, the ME-WE Project, a collaboration across the European Union targeting young carers aged 15–17, focused on three major contributions to research: (1) to systematize knowledge about young carers, (2) to co-design, develop and test, “a framework of effective and multicomponent psychosocial interventions for primary prevention focused on improving their mental health and well-being”, and (3) to carry out wide knowledge translation actions for dissemination, awareness promotion, and advocacy [24]. Survey findings from the project suggest that across all six countries, young carers are taking on significant caring responsibilities for their family members and friends, with reportable adverse mental and physical health effects. The project also found that the co-designed psychosocial intervention positively impacted the educational and employment opportunities for young carers enrolled in the study [24].

Existing scholarship in the U.S. is influenced by the methods and priorities of international research, albeit dominated by the medical and educational sciences. The earliest studies of youth caregiving in the U.S. were undertaken by scholars in the fields of nursing, clinical psychology, pediatric medicine, education, and social work and closely aligned to the health sciences. Kavanaugh et al.’s [6] review of youth caregiving research identified two main trajectories of research in the study of caregiving youth. The first is concerned with describing the caregiving population, including defining age boundaries for ‘youth caregiver’, which can range from under 18 [25] to under 21 [26], and assessing the characteristics of the recipient of care and domestic arrangements such as cohabitation [22,27], including some notation of racial diversity [28]. The second trajectory of scholarship evaluates the impacts of caregiving on youth upon mental health and caregiving capacity [29,30,31,32]. A recent scoping review identified only four primary studies published on U.S. caregiving youth between May 2015 and July 2020 [33]. However, the review parameters excluded studies working with existing large data sets [34]; those taking humanistic, philosophical, or anthropological approaches [35]; non-article-length publications [36]; and the health humanities [37]. In countries where terms and definitions are not systematized or widely used, as in the U.S., comprehensive reviews are stymied by the capacity to identify conditions of caregiving by young people in the absence of signaling terms or keywords in existing research.

### 3.1. Rethinking Recognition

Within the research-policy praxis that makes up much of the scholarship related to caregiving by young people, there is an inherent assumption—and some evidence—that social, political, and economic recognition of youth as caregivers can have beneficial outcomes on psychological wellbeing [25,32], on caregiving competency [38,39], and in exceptional moments of crisis [31]. In most of these studies, recognition is understood as an identification process, suggesting that services and interventions could be directed toward youth caregiving families. Recognition is also broadly conceived as public awareness that could bolster understanding and programming (e.g., [3]).

Recognition also comes with an assumption of non-recognition, and thus is often a solution proposed for populations that appear to fall outside of existing protection or services. Parr [40] explains that populations considered ‘hidden’ in medical and health environments are often excluded from the benefits of public health interventions due to the assumptions and biases that become institutionalized into all aspects of society, including public health. To be a hidden or underrecognized population in public health might be linked to the historical and social construction of people whose intersectional identities disproportionately subject them to injustice [41].

In the case of young people who are caregivers, the assumption that the population has been ‘hidden’ from caregiving or public health legislation suggests the need for recognition, but this can also be a challenging endeavor that requires a degree of epistemic humility. Aldridge [42] cautions that forms of recognition must be treated carefully since categorization can also lead to homogenizing caregiving youth as problematically parentified or uniquely different from other youth (see also [37]). A different caution is offered by Lewis [43], who found that U.S. young adult caregivers were ambivalent about being recognized for their caregiving and uncertain of how it fits with other aspects of race or income. Evans [44] points to the different ways that youth globally must navigate their caregiving identity in relation to other identities including class and race and the ways that these become constructed in and through everyday and national politics. A feminist geography approach to recognition, elaborated by Staeheli [45], reinforces the notion that much of political life occurs outside of legislatures and courts, and so recognition of a population or their conditions must take into account the public and private spaces in which any given group can work in and through social and political relationships in order to influence power.

With this feminist understanding of recognition, we wish to offer an alternative for how we account for recognition of young carers in countries or communities that lack discrete legislation or political identification. In situations of exclusion due to subordinated intersectional identities, recognition can be an ethically consequential act that reorders the distribution of power and resources in society [46]. This is true for the resources required to receive and provide good care, including but not limited to access to housing, food, education, and so on. These needs can be framed as basic human rights that require formalized mechanisms to enforce them. In case of caregiving youth and other hidden populations, it is also important to consider where and when the interactions between justice-centered recognitions, such as the formalization of children’s rights, compare or interact with functional or responsive forms of recognition that emerge in the absence of universal rights. A political analysis of recognition from the position of feminist theory would require more attention to informal, incremental, and incomplete practices and processes adjacent to or outside the formal politics of states.

We draw upon theoretical frameworks of intersectionality and care, particularly as it has been advanced within feminist geographies of health in analyzing progress towards youth caregiver recognition. Geographers approach questions of health and care with particular attention to how place, space, and scale are interwoven with health outcomes and the processes that determine wellbeing. This geographic view considers practices and processes of care as situated “in particular social, economic, and political circumstances—varying over time and space” [47] (p. 4). We add to this framework an understanding of the child as socially, politically, culturally, and economically structured in place and time [48]. Recognizing a child as a caregiver can be disruptive in groups, communities, or societies that are invested in viewing the child only as a care recipient or contributor to miscellaneous domestic work (e.g., [49]). As the political and legislative priority given to public health is weighed against and alongside other national priorities, such as economic productivity, the definition of child as caregiver changes. A full account of the recognition of caregiving youth could thus include attention to the processes that occur outside of and beyond the scope of formal state power, those factors which help to challenge existing assumptions about children and care, and the kinds of relationships that facilitate the reconstitution of youth as caregiver or care recipient in political institutions and definitions.

The U.S. is an important case to consider when appraising how we evaluate and assess recognition of caregiving by young people. In contrast to national strategies to support young carers in other countries, the most ambitious form of support for caregiving youth in the U.S. historically relies on civil society, non-profit organizations, and researchers working in and with geographically bound communities. These relationships emerge from research–practitioner partnerships, build collaborations with states and educational units, and in some cases, provide direct services. The resulting landscape is a vibrant but uneven geography of support and intervention for caregiving youth in the United States, whose recognition and access to services may depend less upon the urgency of their need than on where they live, the multiple vectors of precarity that their families endure, and the specificities of illness or disability that requires care. Furthermore, this uneven geography of care provision exists within a population that is nearly five times larger than the United Kingdom and thirteen times larger than Australia, and a federalist system that leaves the administration of core services ranging from healthcare to education to the determination of states.

For the remainder of this article, we hold together the two somewhat discrete but interrelated problems of (a) analyzing the recognition of caregiving youth in the United States as a public health problem, and (b) describing the patchwork geographies of support and understanding which emerge in the spaces of this absence. Our goal is to illustrate how a focus on the relationships of recognition, both in the past and the present and at local and national scales, reveals a different perspective on caregiving youth in the U.S. with a much more complex history that is also much more integrated into national priorities than previously identified. In line with a critical feminist geography approach and a care ethics emphasis on relationality, we pay special attention to the question of how this hidden population moves into and out of arenas of formal legislation and public health priority. We also attend to relationships established in the absence of policies, sometimes with the goal of building more formal recognition, and other times with the goal of changing practices in a specific location. We take a historical approach to the question of recognition, because alongside the patchwork geographies of recognition, there are also patchwork temporalities, or historical changes in the relationship between formal and informal recognition of young carers.

### 3.2. History of Recognition in the U.S.

To tell a more complete story of caregiving by young people in the contemporary U.S., we reflect on three critical moments in U.S. political history when people under the age of 18 were either recognized as caregivers, or restricted to the role of care recipient: the Little Mothers program of the early 20th century, the Family Medical Leave Act (FMLA) of the early and late 20th century, and the social movements to recognize family caregivers and caregiving youth in the first quarter of the 21st century. Together, these historical moments reveal that recognition of youth as caregivers travels in tandem with public health research, social movements, and national priorities associated with political status and economic security. They are selected because they also touch on three important moments of the coupling of public health with national priorities, including the emergence of public health as both a discipline and strategic priority in the early 20th century, concern over a diminished female workforce at the end of the same century, and the anticipated caregiving crisis of the early 21st century. Here, we briefly describe the program or policy and the ways that young people become recognized or hidden as caregivers. We suggest that though the rights of children who are caregivers are not evident in policy or legislation, they become increasingly important for practitioners, researchers, and activists in the early 21st century, resulting in a type of recognition which, in its blending of rights and economic logic, is unique to the U.S. context.

#### 3.2.1. Little Mothers

In 1911, immigrant ‘Little Mothers’ of the nation’s largest cities were acknowledged and promoted as lifesaving caregivers for their infant siblings when their mostly immigrant parents struggled in the poor conditions of work and poverty [49]. As part of the broader Save the Babies movement to reduce national infant mortality rates, Little Mothers were targeted by public health reformers to learn how to keep babies well. Olson [37] explains how these efforts intersected with changing ideals about domestic work for both women and children, as well as eugenics and vigorous disagreements about who might become part of the future of the nation.

The recognition of young people as caregivers in this era was driven by concerns of the newly professionalized public health practitioners working in and around large U.S. cities, and a global rush to reduce infant mortality rates [50]. Dr. Josephine S. Baker, who was previously known for her identification of Mary Mallon as the source of an outbreak of Typhoid in New York in 1906, focused her work in New York City tenements on the environmental conditions producing high infant mortality rates [51]. In addition to identifying the urgent need for milk stations, Dr. Baker elevated the profile of the children who were tasked with caring for their infant siblings while their immigrant parents worked long hours. She advocated for the design of services focused not on the wellbeing of the young caregivers, but on their skill and capacity to keep their infant charges healthy and alive. This work resulted in the Little Mothers’ Leagues, groups organized and run by public health officials, women’s leagues, and the burgeoning population of graduates of the new discipline of social work. Little Mothers’ Leagues consisted of meetings and a curriculum designed to help children and adolescents keep babies well, with instruction on cleanliness, food, sleep, and tending to general environmental conditions.

The Little Mothers’ Leagues spread across the U.S. through the 1920s, but several forces shifted it away from a focus on children providing lifesaving care for siblings and towards a model of home economics training in which young girls were to be trained to provide care for their future children. As late-19th century European immigrants were more fully absorbed into the U.S., and as family care was increasingly shifted into the private space of the home, the recognition of young people as lifesaving caregivers was replaced by a national imaginary of children as care recipients whose childhood should not be burdened by care responsibilities—except, that is, for those who would become parents in a future time [37].

There are two key characteristics of the case of the Little Mothers origins as a program for young caregivers, and its eventual displacement by an educational emphasis on preparing the girl child to be a future mother. First, it was motivated by a national and international concern with infant mortality rates, and therefore did not focus on the wellbeing of the Little Mother herself. In fact, there is evidence in the records of some early social workers that they considered the Little Mother to be a kind of ‘lost cause’ who was viewed only slightly more favorably than her immigrant parents but was seen primarily as a means by which her younger siblings might be integrated into the future of the nation. This contrasted with social movements demanding that children be removed from dangerous labor conditions, motivated by an image of childhood in the U.S. that included healthy play and compulsory education. Poor, racialized, and ethnicized, the immigrant Little Mother was excluded from this new childhood social imaginary. Second, the attention to sibling caregivers marked the only time in the 20th century in which young people who provided informal care to family members were recognized as an important element of a broad public health agenda related to family health and wellbeing. It coincided with arguments that favored the domestication of caregiving as work undertaken by women in the home, a factor that explains the slow emergence of federal attention to the rights of caregivers; it was not until white women, who also had family caregiving responsibilities, entered the workforce en masse through the 1960s and 1970s that caregivers rights became a subject of concerted political effort and attention in the form of the Family Medical Leave Act.

#### 3.2.2. The Family Medical Leave Act (FMLA)

The Family Medical Leave Act (FMLA) of 1993 enabled people employed by public agencies, including local, State, and Federal employers, and local education agencies (schools), and also by private sector employers who employ 50 or more employees, to take up to 12 weeks of unpaid leave in a 12-month period, retain access to employer-provided health insurance and retain their position or an equivalent job upon return to work. Eligibility for the FMLA is focused on care responsibilities with eligible life events, including the birth of a child or the adoption or foster care of a child and to care for the newborn child or newly placed child; to care for self or spouse, child, or parent who has a serious health condition; and care for a family member who is in active military service when the servicemember acquires a serious injury or illness [52].

Although the FMLA is often framed as a needed solution to family caregiving, it was, more accurately, a work and labor bill that emerged as a response to activism and mobilization of a civil rights agenda embraced and advanced by women’s rights movements who recognized that an antidiscrimination approach would be more successful than a focus on maternity leave or the positive rights of women caregivers [53]. Representative David Bonior, then Democratic Whip, articulated the resulting gender neutrality and antidiscrimination sentiments of the legislation ahead of its passage, commenting that “Parents should not be asked to choose between their jobs and caring for a sick child…” [54]. Originally introduced in 1984, the congressional act reflects the growing reliance on women in the workforce to ensure national economic productivity. Its central provisions were designed for employees to take leave to attend to the reproductive demands that typically fell to women (see also [13]) while retaining their health insurance and without jeopardizing their position at work or threatening national growth. Thus, the celebration of the bill and its enactment into law was welcomed as a needed protection for working women and for some segments of the caregiving population, but its broader conception was a strategic political move to link it to civil rights.

The outcomes of the FMLA have been criticized from a range of different issues and perspectives. The legislation’s emphasis on preserving work potential outside of one’s home meant it did not address gender or racial inequality in the workforce and at home [55,56,57]. The legislation did not meaningfully reflect the longstanding fight to have the domestic work of women recognized as gainful work (e.g., [58]), or the historical differentiation that race has played in conceptions of labor, family, and care (e.g., [59]). As a bill primarily focused on the responsibilities of employers, the FMLA did not carry scope or capacity for addressing caregiver needs beyond formal workplaces. These critiques were in addition to others that suggested that even for the intended population, the law has been criticized for not requiring paid leave, having limited eligibility, and being difficult to enforce, leaving workers unprotected [54]. Talley and Crews [12] suggest that caregiving was only beginning to emerge as a public health problem in the 1980s, and so the population-wide impacts of informal caregiving were still relatively fresh in both research and policy. In summary, the FMLA had shortcomings both as a labor bill intended to protect caregivers who worked and as a legislation intended to protect the rights or wellbeing of caregivers. However, it was also an important moment in which caregivers’ rights were acknowledged and protected in a national agenda.

The FMLA is relevant to the history of caregiving youth recognition for many reasons, but we highlight two areas of significant impact. First, the FMLA established family caregiving as a work problem that had to be solved for full-time employees. This boundary meant that young people under the age of 14 in most U.S. states, and even those under 18 in many U.S. states, would be ineligible for protections given the legal limitations on the total hours of employment they are allowed to work. Nor did it engage questions of civil rights for full-time students engaged in care, or any federal or state requirement for the protection of their rights in education. Second, and relatedly, the legislation had a lasting impact on the legal and social imaginaries of the age of family caregivers. Both the social movements surrounding the emergence of the FMLA, and the political rationale attributed to the need to protect workers from unfair dismissal, perpetuated the normative standard of a caregiver as a woman, usually a mother, caring for children under the age of 18 or for dependent adults over the age of 18. The assumption that family caregiving is domestic work undertaken by people over the age of 18 continues into the 21st century in even the most important scientific evaluations of the state of caregiving in the U.S. (see, for instance, [15]).

It is important to emphasize, once again, that the FMLA was a work bill and not intended to resolve the kinds of long-term care problems that come into focus in the 21st century, with the overall aging of the U.S. population and the decline of the caregiving labor force. This is perhaps most evident when compared with the Older Americans Act of 1965, which created provisions for support of caregivers articulated by Title III of the OAA. Today, the resulting infrastructure of Title III includes mandatory state structures for supporting both older people and their caregivers, including but not limited to the Area Agencies on Aging that provides resources ranging from respite resources to support and programming through federal support [60]. In contrast to the OAA, which provided opportunities for caregiving to be framed as a problem of public health and wellbeing of both caregiver and care recipient, the problem of family caregiving under the FMLA was framed as protection of the civil rights of those who are employed and having their work interrupted by caregiving for a discrete period of time.

Against the progressive era impulses of communal public health that drove the Little Mothers programs earlier in the century, the FMLA suggests a shift by both activists and politicians away from public health goals, and towards civil rights and fairness in work, labor, and economy. However, by the early 2000s, demographic changes and late neoliberalism were already exposing the limits of work-focused legislation for caregivers as the nation entered a new squeeze on informal care. When first Lady Roslyn Carter [61] published an article titled “Addressing the Caregiving Crisis”, the complexity of family caregiving in the contemporary U.S. was approaching a tipping-point. Women were increasingly caring for both aging parents and children while working full-time; aging adults were living longer, as were family members with illnesses that might have been fatal at the turn of the 20th century; institutionalization was being replaced by ‘aging at home’; and the domestic workforce, long supported by low-income women of color and immigrant women, was declining under the pressure of poor wages and undesirable work conditions. Care ethicists such as Joan Tronto [62] and others captured a quiet following by those who agreed that crises of care were not one only about the civil rights of women in the workplace, but the undervaluing of care in political, social, and economic life. Still absent was a recognition that young people might be caregivers for their families, rather than just care recipients.

#### 3.2.3. New National Agendas and Patchwork Geographies of the Early 21st Century

By the beginning of the 21st century, the need to focus on family caregivers in the U.S. found new momentum through research and advocacy. Gail Gibson Hunt founded the National Alliance for Caregiving (NAC) in 1996, and as the organization grew into a national advocacy and research network, it focused on making visible both the interpersonal and the economic contributions of family caregivers in the U.S. The 1997 study [63] conducted by American Association for Retired People (AARP) and NAC, *Caregiving in the U.S*., established a benchmark for measuring and recognizing the reproductive work of families who were caregiving for adults. It reflected new attempts by researchers to identify the direct contributions of reproductive caregiving labor by estimating the economic value of informal care work [64]. *Caregiving in the U.S.* was repeated in 2007, 2009, 2015, and 2020, revealing declining health and increased pressures, longer hours of caregiving for more years by a more racially diverse population. Through the work of disease-specific, aging-focused, and care-focused non-governmental organizations, the care crisis was increasingly being framed not only as a shortage of quality care or disruptions to national productivity, but also as a threat to the wellbeing of the caregivers themselves.

Mainstream organizations were still not including young people as caregivers in research or policy solutions in the earlier discussions of the care crisis, but awareness was growing as some scholars and leaders in the caregiver wellbeing movement acknowledged that in families with complex care needs, children and adolescents were important secondary caregivers, and even primary caregivers. A real change in recognition for caregiving youth began in 2005, when the NAC and the United Hospital Fund produced the first report on young informal caregivers. *Young Caregivers in the U.S.* [22] provided evidence of the oft-cited figure of 1.3 to 1.4 million family caregivers under the age of 18 providing support for their families. It also serves as a record of the network of researchers, practitioners, and advocacy around young people as caregivers that had not previously been supported in the U.S. Several of the authors and advisors on the report, including Gail Gibson Hunt, Carol Levine, and Dr. Kim Shifrin continued advocating or providing evidence for caregivers under 18.

In the fall of 2006, Dr. Connie Siskowski, who was also an advisor on the report and Founder and President of Boca Respite Volunteers in Boca Raton, Florida, initiated the first in-school support program for caregiving youth in the U.S., in partnership with the School District of Palm Beach County. Dr. Siskowski, whose doctoral work at Lynn University had focused on young people who were caregivers, shifted the work towards caregiving youth and the organization became the American Association of Caregiving Youth (AACY) in 2010, with a mission to extend awareness and support nationally, while also building the Caregiving Youth Project to serve more young people in Palm Beach County. During the years immediately before and after the NAC/UHF study, Dr. Siskowski also contributed to the research evidence of impacts of caregiving on student education [1], hospice and home healthcare [65], Latino youth caregivers [29], and drawing attention to health-related concerns [21].

Though much of the direct service provided by the CYP was focused on Palm Beach County and raising awareness through the work of the AACY, the organization has also served as a meeting place for interdisciplinary researcher and practitioner collaborations. In Florida, for example, Dr. Donna Cohen [25] and Dr. Julia Belkowitz [27] contributed early studies and student research collaborations through the disciplines of Psychology and Pediatric medicine, respectively. In 2015, Dr. Elizabeth Olson brought researchers and policy actors to the University of North Carolina at Chapel Hill to create new networks, which resulted in the establishment of the Caregiving Youth Research Collaborative (CYRC), directed by Drs. Olson and Siskowski, to advance the impact of research and evidence to benefit caregiving youth and their families. The work of the CYRC shifts as research and policy changes provide new opportunities for influence. At the time of writing this article, the CYRC is focused on producing a ‘state of the field’ report on caregiving youth in the U.S. to be released in Fall 2023. This model of researcher–practitioner partnership is an important characteristic of the strides made in the U.S., and it explains the geographic patchwork of awareness, evidence, and practices. The size and scope of the family caregiving population in the U.S., its diversity and heterogeneity across different regions of the country, and the influence of state autonomy and authority even within federal policy translate into an unevenness of impact and action, with bright points of engaged activity, such as the Caregiving Youth Project in Palm Beach County.

Recognition of caregiving youth has also occurred through researcher-led initiatives. Partnerships with national disease associations to advance research and with a focus on key populations of caregiving youth with high levels of responsibility, as illustrated by Dr. Melinda S. Kavanaugh’s work out of the University of Wisconsin in Milwaukee, through local, national, and global research and program development with an emphasis on children caring for family members with neurological disease [66]. Increasingly, partnership with state education agencies is leading to advances in research and recognition due to state control of and responsibility for education. For example, a single question developed for the Youth Risk Behavior Survey by Dr. Siksowski and Dr. Olson, approved by the CDC, was administered for the first time in the state of Florida in 2019 [67,68]. The question posed, “During an average week, on how many days do you provide care for someone in your family or household who is chronically ill (lasts 3 months or more), elderly, or disabled with activities they would have difficulty doing on their own?”, came with the option to select “none”, “1–2 days”, “3–5 days”, or “6–7 days”. The single-item question has been adopted subsequently by the state of Colorado for its 2023 Healthy Kids school-based survey. A different model of collaboration was embraced by the Rhode Island Department of Education (RIDE) after learning about the significant amount of both caregiving and paid work undertaken by students (see [68]), which has approved the first curriculum in the U.S. directing all schools to support caregiving youth and will be supported by the AACY and resources developed by researchers and practitioners, including that drawn from the successes of the Caregiving Youth Project.

This patchwork geography of recognition of caregiving youth, growing in both scope and reach, was in place when The Recognize, Assist, Include, Support, and Engage (RAISE) Family Caregiving Act was signed into law in January 2018 [69]. RAISE establishes national strategies for family caregiving by convening diverse stakeholders who focus on care. The Act includes three major elements: (1) the formation of the nation’s first Family Caregiving Advisory Council; (2) the development of a Report to Congress; and (3) the creation of the National Family Caregiving Strategy. The resulting 2022 National Strategy to Support Family Caregivers (NSSFC) both removes the age bias, and even highlights the shift as a deliberate adjustment intended to include children and adolescents:

The RAISE Family Caregivers Act defined “family caregiver” as “an adult family member or other individual who has a significant relationship with, and who provides a broad range of assistance to, an individual with a chronic or other health condition, disability or functional limitation. In its initial report to Congress, the RAISE Advisory Council expanded that definition slightly to include unpaid individuals of all ages in its definition.

[69], (p. 7)

In addition to removing the age bias that institutionalized caregivers as only adults in previous federal legislation and policy, the 2022 NSSFC identifies “caregiving youth” as a priority population of special interest that is underserved or difficult to reach [69], (p. 33) and includes references to the needs of caregiving youth throughout the document, with references to educational professionals and employers.

The inclusion of youth caregivers in RAISE and the new National Strategy reflects a new era of federal, formalized recognition for people under the age of 18 as well as our current moment in the caregiving crisis. With the increasing attention to the shifting demographics as more Americans enter older adulthood, and more broadly, the widening gap between the number of people who require caregiving and the availability of both professional and family caregivers [61], this crisis precipitates certain types of responses, including bringing more people into the category of informal caregiver. As in the examples drawn from the 20th century, a contemporary public health concern reconfigures the role of young people in the larger dynamics of labor force availability and recognition of caregiving. How this recognition translates into changes in practices remains to be seen, but the inclusion of President Biden’s 18 April 2023 Executive Order on Increasing Access to High-Quality Care and Supporting Caregivers of “minor children” as people who are essential to national wellbeing by providing informal and mostly unpaid care suggests that the framing of young people as caregivers has established itself in the highest levels of government [70].

## 4. Discussion

In each historic example, a contemporary public health concern coincides with movements and societal changes that establish a role for young people in the larger dynamics of labor force availability and recognition of caregiving. Young people as caregivers surfaced in the early 20th century to address a perceived absence of domestic responsibility in working poor immigrant households where adult family members were needed to ensure factory productivity. The FMLA emerges with the movement of women into the workforce and their activism for greater recognition and equality. However, rather than focusing on the wellbeing of women as caregivers, advocates strategically mobilized arguments for the civil rights of all workers antidiscrimination. Unlike the Little Mothers League, the FMLA approached caregiving responsibilities as explicitly adult activities, most frequently predicated on the presence of a child who needs care.

The denial and subsequent invisibility of youth caregivers in the U.S. policy landscape has only recently been interrupted by the reconfiguration of care work across productive and reproductive spheres and organizations under pressure of an impending crisis. By the 21st century, the conditions of care availability, both formal and informal, become framed as a threat to economic stability against the growing demands upon informal caregivers, and the very real public health threat presented by not having enough informal or paid caregivers to support an aging population. The crisis of care opens the possibility for both the productive (neoliberal capitalism) and reproductive (the value of care) logics to establish themselves in federal policy. The mismatch in the demand for care labor and shortage of care workers, combined with the researcher–practitioner partnerships that have yielded evidence of previously unaccounted for caregiving by young people, provides the foundation to consider the role of young people in household caregiving configurations.

These moments illustrate how important it is to understand recognition of ‘hidden’ populations in public health as happening—and perhaps, changing—across time and space. They interweave different outcomes of caregiver recognition, gender relations, labor and economic organization, social movements and advocacy networks, and disciplinary and research advances created contexts that conditioned the options available for recognition of caregiving youth. The result is the figuring of young people as either caregivers or as recipients of care as they are incorporated into broader strategies intended to improve public health and economic outcomes. Our longer historical framing suggests that the U.S. was early in recognizing the need to support young people who provided care to family members, but young people were then ‘hidden’ through much of the 20th-century debate about how caregiving mattered to the nation. As Tronto [71] has cautioned, the U.S. has largely failed to conceive of a democracy that centers care and its value in the reproduction of households and society. Even as scholars try to translate the value of informal care into the dollars and cents of the formal economy, it is unclear whether these arguments will be convincing enough to instigate the dramatic restructuring that a care-centered political economy would require. Each of these moments nonetheless represent an imperfect valuing of care: the first through an investment in a future nation through public health investments; the second, by creating space for adult caregivers in the workforce; and the third, to value caregiving directly in order to buttress a system under crisis.

As both a public health history and a geography of the political response to care by young people, our analysis also suggests that evaluations of recognition could be improved if we pay more attention to the scales and places that form a geographic patchwork that will determine future directions in research, practice, and policy [72]. The United States has made substantial progress in the formal recognition of caregiving youth by acknowledging children and adolescents as providing informal care to family members and others, and this progress has the potential to facilitate more funding for both research and support for caregiving youth and their families in line with more advanced nations [3]. Nonetheless, being attentive to geographic and historical variation could be especially important for large, populous nations, or those with a high degree of decentralized power in areas of health and education provision and policy. For practices of public health, looking for this patchwork geography of high recognition and engagement, or networks that create new geographies of caregiving youth support outside of and beyond the state, could present options for new best practices in the absence of a federal rights framework.

## 5. Conclusions

Over the past century, people under the age of 18 surface and then are again hidden as caregivers in the view of federal policy in the U.S. Partly as a consequence, the U.S. has been considered by experts to be substantially behind other nations in the support of young carers. In this article, we point to historical factors that determined whether and if a child is primarily framed in federal and state policy as a care recipient, a caregiver, or both. Though scholars have emphasized the formal rights assigned to children as a signal of potential recognition of young carers, we find that, in the U.S. context, historical and contemporary inclusion of caregiving youth in coordinated public policy is based less on rights-based arguments, and instead is successful when linked with economic and public health priorities, alongside evidence-based arguments for including young people in policy responses.

The case of the U.S. suggests that even if acknowledgement of the rights of children is good and defendable for many reasons, it is not necessary to bring about recognition for young people who are carers. In each era of care legislation, we note different values—those of public health, the economic pressure of the care crisis, or even new research collaborations—that open the space for young people being recognized and included in care policies. The current momentum towards recognition emerges from research–practitioner partnerships which made young people (under 18) legible as family caregivers. The institutionalization of this shift was supported by government and non-government actors armed with diverse research and examples of successful interventions to both raise awareness and encourage inclusion. Researchers and practitioners seeking recognition for young carers in similar contexts might find some inspiration in the progress towards recognition in the U.S., while learning from models of longer-established recognition in other countries.

This present analysis provides a historically nuanced understanding of the construction of caregiving youth by actors and institutions with the power to enhance or limit recognition. However, it is still limited to those processes that can be identified through legislation, formalized service providers, and discernable policy outcomes. Our ongoing research on the everyday geographies of caregiving youth (Youth Family Caregivers and the Geography of Childhood, National Science Foundation award number 1853260. Elizabeth Olson, P.I.) suggests that there is another way to approach these questions from a feminist geography of health at the scale of community. We are learning how caregiving youth create, envision, and mobilize when trying to care for themselves, their loved ones, and their communities [73]. Even with formal recognition, caregiving youth in the U.S. face housing and food insecurity, chronic poverty, and poor access to healthcare. In these new stories which center young people’s everyday lives both in and beyond their care obligations, we hope to extend our understanding of what kinds of recognition might be needed to address these systematic pressures placed upon caregiving youth in the U.S., and the complex care needed by the families that they support.

## Data Availability

Not applicable.
